# Analysis of breathing patterns to stabilize cardiovascular changes in physical stress environments : inspiration responds to rapid changes in blood pressure

**DOI:** 10.1007/s13534-024-00379-y

**Published:** 2024-04-10

**Authors:** Daechang Kim, Seungbin Baek, Seunghui Kim, Sanghee Im, Sungmin Kim

**Affiliations:** 1 Department of Medical Biotechnology, Dongguk University, Bio Medi Campus, Ilsandonggu, Goyang si, Gyeonggi-do 10326 Korea; 2 Department of Medical Device Business, Dongguk University, 32, Dongguk ro, Ilsandonggu, Goyang si, Gyeonggi do 10326 Korea; 3 Department of Regulatory Science for Bio-Health Medical Device, Dongguk University, 32, Dongguk ro, Ilsandonggu, Goyang si, Gyeonggi do 10326 Korea; 4 Department and Research Institute of Rehabilitation Medicine, Severance Hospital, Yonsei University College of Medicine, 50-1 Yonsei-ro, Seodaemun-gu, Seoul, 03722 South Korea; 5 Department of Medical Device Industry, Dongguk University, Seoul, 04620 Korea

**Keywords:** Autonomic nervous system, Physical stress, Breathing patterns, Cardiovascular system

## Abstract

The thoracic nerves form a complex neural network that coordinates involuntary muscles such as breathing and the heart. Breathing has various patterns to maintain homeostasis in the human body. This study analyzes changes in the cardiovascular system and breathing patterns induced by stress caused by various mechanical movements performed in daily life and ultimately, the goal is to propose effective breathing patterns and breathing control methods to maintain cardiovascular homeostasis. The participants’ age was 26.97 ± 3.93 years, height was 170.24 ± 8.61 cm, and weight was 65.69 ± 13.55 Kg, and there were 62 men and 38 women. Breathing and electrocardiogram were obtained using HiCard+, a biometric monitoring device. The measured electrocardiogram was analyzed for heartbeat interval, which indicates changes in the cardiovascular system, and standard deviation of normal to normal interval (SDNN) and root mean square of the successive differences (rMSSD), which indicate the activity of the autonomic and parasympathetic nervous systems. For respiration, time changes were analyzed as patterns by calculating inspiration and exhalation times. As a result of this study, rapid changes in blood pressure increased SDNN and rMSSD from 0.053 ± 0.06 and 0.056 ± 0.087 to 0.109 ± 0.114 and 0.125 ± 0.170 s, and induced an increase in spontaneous inspiratory time from 1.46 to 1.51 s (*p* < 0.05). Ultimately, we hope that the results of this study will be used as a breathing control training technique to prevent and manage rapid cardiovascular changes.

**Supplementary Information:**

The online version contains supplementary material available at 10.1007/s13534-024-00379-y.

## Introduction

Breathing is a key function of the body for maintaining a healthy life. The function of breathing is to inhale oxygen, which is used as fuel for metabolic activities and expel carbon dioxide, a byproduct. These functions are performed by the respiratory system and are coordinated by the thoracic nerves, a pair of spinal nerves that emerge from the thoracic region of the spinal cord. The thoracic nerves form a complex network of nerves that communicate with each other and coordinate the respiratory muscles used for breathing. Additionally, it can interact with the autonomic nervous system (ANS) to regulate involuntary muscle functions such as heart rate and digestion [1–3]. The respiratory system induces a breathing pattern in response to environmental changes inside and outside the human body that occur during walking, repetitive exercise, or mechanical movement. These breathing patterns appear through numerous interactions between the ANS to maintain homeostasis, and thus have a wide variety of patterns [4, 5].

The most common disorder that can arise from breathing patterns is dyspnea [6]. Dyspnea is caused by feeling uncomfortable breathing. In the case of the elderly, 36% of the total elderly population suffers from dyspnea, and a total of 3.4 million outpatient treatments are performed annually. Importantly, 44.6% of patients who visited due to dyspnea were also found to have life threatening cardiovascular diseases such as acute heart failure and arrhythmia [7]. These causes include increased spasticity of the chest wall, decreased mechanical function of the respiratory system such as elasticity and recoil of the lungs, and decreased efferent nerve responses of respiratory muscles due to low oxygen pressure [8, 9]. To solve these problems, methods using mechanical and psychological therapies such as stretching, meditation, and breathing pattern exercises are being used, and research is being conducted to prevent and treat diabetes, high blood pressure, oxidative stress, and breathing dyspnea [10–16]. The biggest point is that breathing control training can be used as a practical management method for prevention and intervention in everyday life, home, and work environments in the control of various disorders, and mental stress [17, 18].

For this reason, the importance of breathing in the interaction between the respiratory and cardiovascular systems is increasing. The interaction of these systems occurs in the physical stress caused by various movements, and homeostasis is maintained through the ANS and neural networks [19–21]. One of these reactions, respiratory sinus arrhythmia (RSA), is a physiological phenomenon that represents a change in heart rate that occurs simultaneously with breathing and reflects the interaction between the respiratory and cardiovascular systems [22–24]. RSA is reflected to a greater extent by spontaneous breathing. Spontaneous breathing is a reaction of the respiratory system produced by involuntary muscles, and it has been confirmed that slow breathing can stabilize the function of the cardiovascular system by increasing the activity of the parasympathetic nerve (PN) of the heart [25]. However, there is few research analyzing spontaneous breathing patterns and changes in the cardiovascular system that occur under physical stress caused by various movements in daily life.

Accordingly, we analyzed breathing patterns and electrocardiogram (ECG) changes for the 15 most commonly performed movements in daily life using medical monitoring equipment that can simultaneously measure breathing of the respiratory system and ECG of the cardiovascular system in this study. The final purpose of the study is to analyze the ECG that responds to physical stress that occurs during movement and to confirm the interaction between the respiratory and cardiovascular systems by confirming the breathing pattern that responds to ECG changes. Through this study, we analyzed changes in the cardiovascular system that require spontaneous breathing and proposed a breathing control training method using effective breathing patterns. Finally, we expect that it will be used as a personalized method and digital healthcare technology through additional research.

## Method

### Participant recruitment

The study was conducted with approval 1-2023-0006 through the Institutional review board of Yonsei university severance hospital. Participants were selected as those aged between 20 and 40 who had no discomfort in their daily lives and no history of cardiovascular or mental disease. The total number of clinical participants was 100, and the number of people who missed screening or dropped out was 0. Accordingly, analysis was conducted using data from 100 participants (Table 1). The experiment was explained to all participants, and consent to collection of personal information and use of data for research was obtained through a consent form.

**Table 1:** Clinical trial participant demographic characteristics

Participants (count)	100			
	Male	62	Female	38
Height (cm mean ± std)	170.24 ± 8.61			
Weight (kg mean ± std)	65.69 ± 13.55			
Age (years mean ± std)	26.97 ± 3.93			

### Clinical trial design

As shown in Table 2, physical stress environments consisted of 15 movements commonly performed in daily life, and each movement was performed a total of 5 times. A safe environment was created for all movements, and prior training and explanations regarding falling movements were provided. Each movement performed an average of 10 s, with a 30 s rest period. However, when the participant’s heart rate rose to a high level, a longer rest period was provided at the discretion of the clinical director. The measurement of data for all movements was completed within a maximum of 1 h, and all data was converted into excel data by labeling each movement.

### Data measurement and analysis

**Fig. 1:**
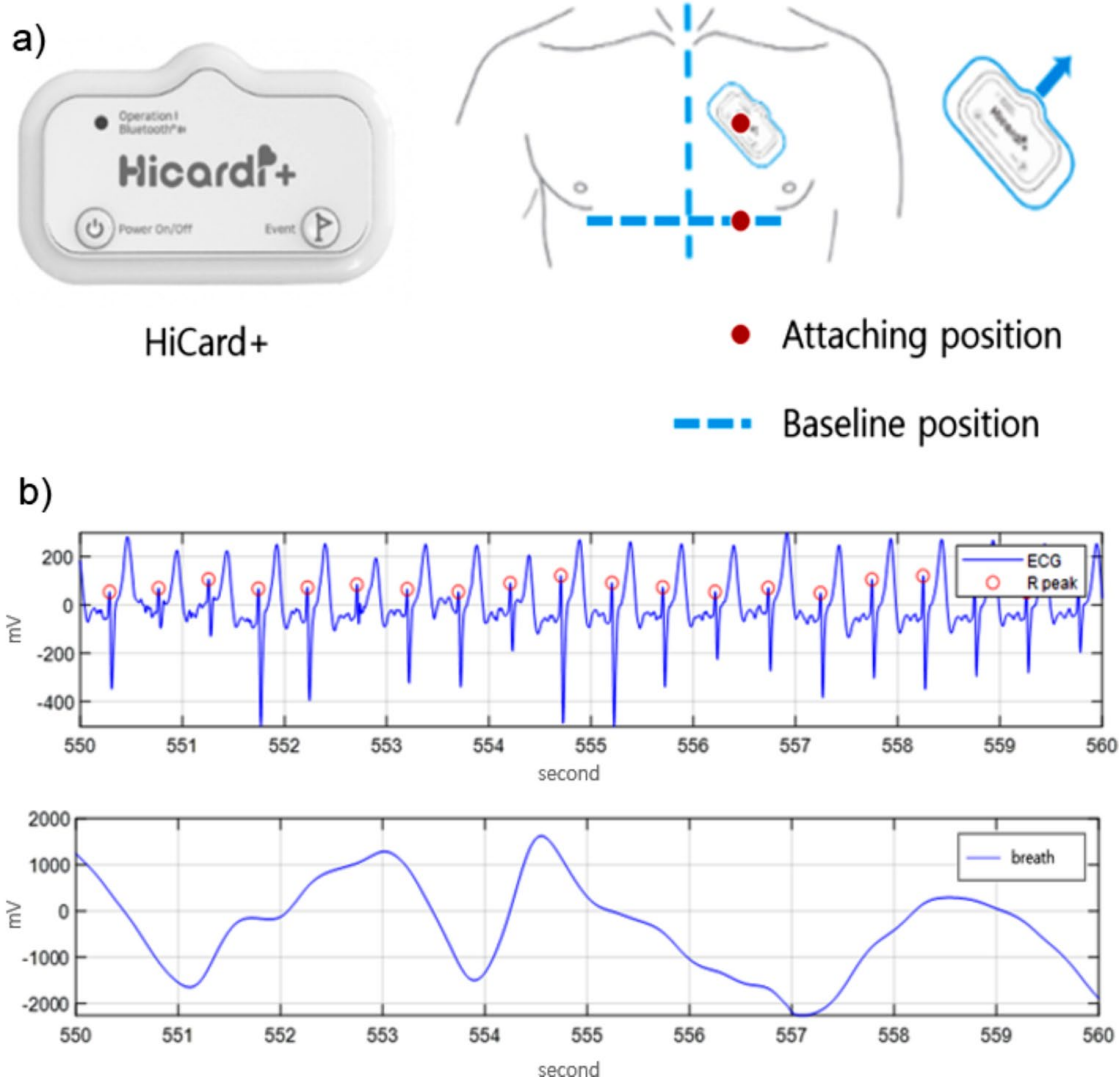
Medical devices used in research. (**a**) Medical monitoring equipment and attachment locations, (**b**) electrocardiogram and respiration actual data using equipment

Mezoo’s HiCard+, which was used to measure data, is a patch type electrocardiogram monitoring medical device and detects the heart’s electrical activity and breathing in real time. The measured data is stored in a cloud database in real time to analyze and detect various biometric information phenomena and provide visual information. The attachment location of HiCard + is as shown in Fig. 1. It was attached to the left side of the chest below the collarbone based on the solar plexus (Fig. 1, a). However, in cases where attachment was difficult, it was attached on the flat left side at the same position as the solar plexus reference line. The final data consisted of a total of 7,500 standardized data sets consisting of 500 for each movement, and the average was calculated, and a t-test was performed to verify the significance of the difference in averages.

**Fig. 2:**
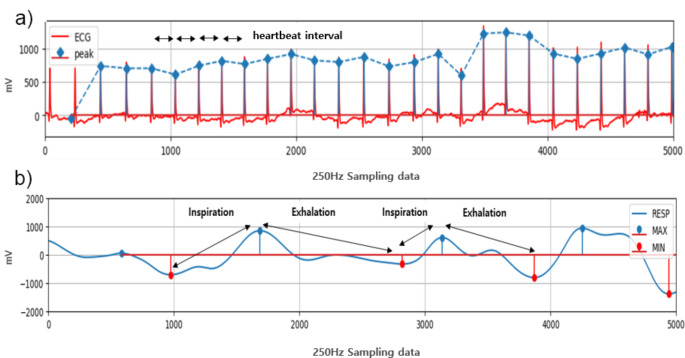
How to derive analysis values using measurement data. (**a**) Obtain heartbeat interval by calculating the time difference between heart beats using an electrocardiogram, (**b**) Analyze inspiratory and expiratory times by acquiring the maximum and minimum respiration positions

To observe cardiovascular system, the 5 s average heartbeat interval of 0 to 5 s and 5 to 10 s at the start of movement was calculated to confirm changes in heart rate. Additionally, standard deviation of normal to normal interval (SDNN) and root mean square of the successive differences (rMSSD), which are time domain analysis methods using heart rate variability, were calculated from the 10 s ECG during which the movement was performed [26–29]. The calculated SDNN represents the comprehensive activity of the ANS, and rMSSD refers to the activity of the PN system, so the patterns of changes in the ANS and cardiovascular system that occur due to physical stress were analyzed (Fig. 2, a).

1 $$SDNN\, = \,\sqrt {{1 \over {N - 1}}\,\sum\limits_{i = 1}^N {{{{\rm{(}}R{R_i} - R{R_{mean}}{\rm{)}}}^2}} }$$

2 $${\rm{rMSSD}}\, = \,\sqrt {{{\rm{1}} \over {{\rm{N - 1}}}}\,\sum\limits_{{\rm{i = 1}}}^{\rm{N}} {{{{\rm{(R}}{{\rm{R}}_{{\rm{i + 1}}}}{\rm{ - R}}{{\rm{R}}_{\rm{i}}}{\rm{)}}}^{\rm{2}}}} }$$

In the case of breathing data, chest impedance data that changes depending on inspiration and expiration of breath was measured. In the case of breathing data, chest impedance data that changes depending on inspiration and expiration of breath was measured. For data loss due to errors, the Nan value was replaced using a moving average. Finally, to remove noise caused by unnecessary movement, preprocessing was performed with a band pass filter in the range of 0.15 to 0.5 Hz using python scipy.signal.butter. The average breathing length for all movements was 3.00 ± 0.07 s. To analyze at least one breath, a total of 20 s of data, including rest time, was divided into four 5 s pieces of data to form breathing data. The highest and lowest points were obtained from the composed data to obtain the respective inspiration and expiration times (Fig. 2, b).

## Result

### Changes in heart rate variability by movement

**Fig. 3:**
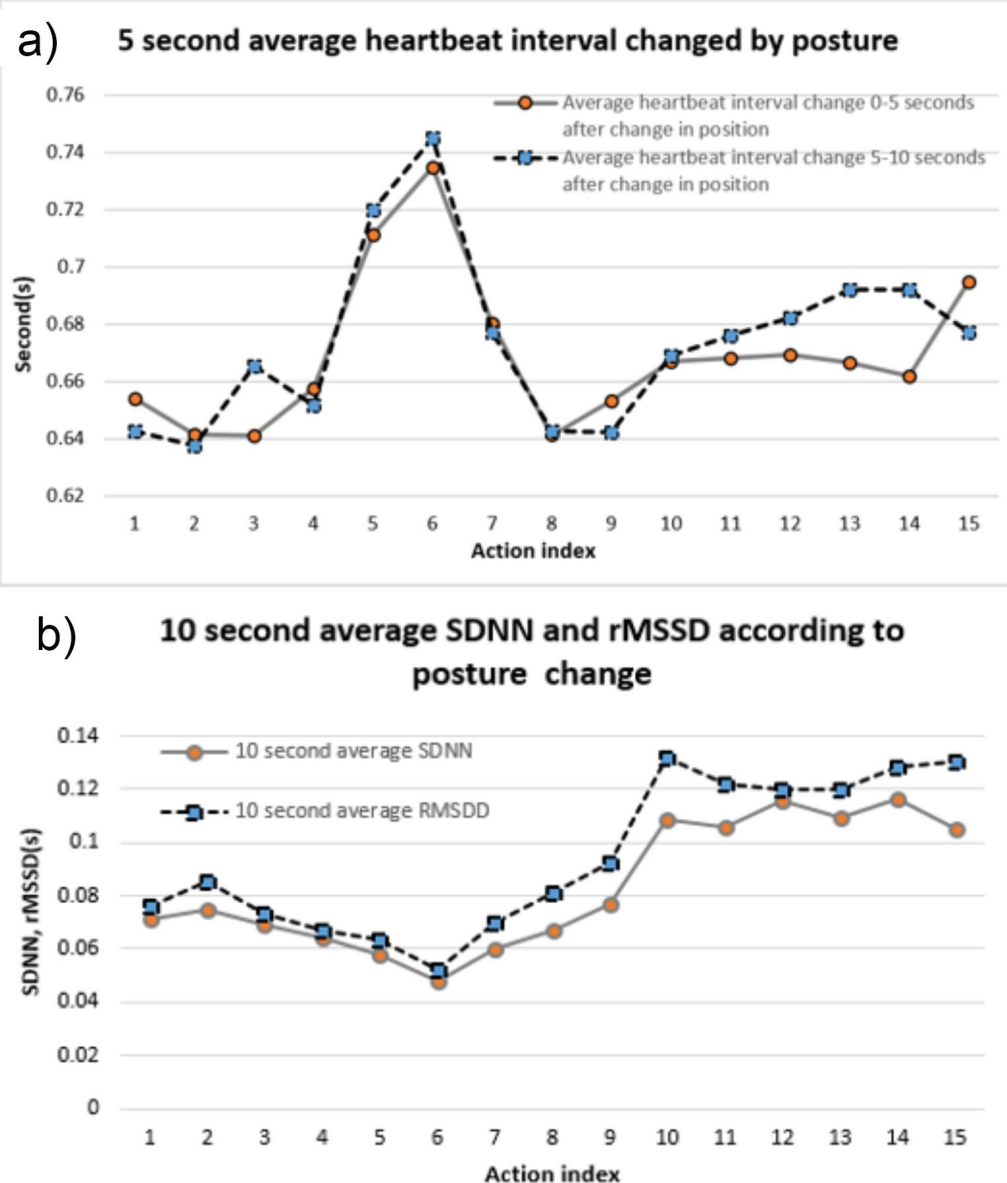
Real time change analysis using electrocardiogram. (**a**) Comparison of 5 s average heartbeat interval, (**b**) Comparison of SDNN and rMSSD for real time mutation confirmation

The average heartbeat interval for 0–5 s was calculated to check the momentary change in physical stress caused by movement, and compared with the average heartbeat interval for 5–10 s to check the changes in the cardiovascular system that occurred thereafter. As a result, it was confirmed that different heartbeat interval changes occur depending on physical stress. The movements that significantly increased heartbeat interval are the index 3, 13, and 14 in Table 2 (*p* < 0.05). Each heartbeat interval increased from 0.64 ± 0.13, 0.66 ± 0.17, and 0.66 ± 0.16 to 0.66 ± 0.13, 0.69 ± 0.15, and 0.69 ± 0.15 s (Fig. 3, a).

In the results of SDNN and rMSSD to confirm changes in the cardiovascular system and ANS, the lowest values were confirmed at indices 5 and 6 of Table 2, which generally include rest type movements with low physical stress. In stair walking (index 1, 2 of Table 2) and exercise movements (index 7, 8, 9 of Table 2) that induce physical stress, SDNN increased to 0.073 ± 0.083, 0.067 ± 0.10 s (*p* < 0.05), and rMSSD increased to 0.080 ± 0.116, 0.081 ± 0.150 s (Fig. 3, b). All related changes showed a significant value of *p* < 0.05. These results show that it is possible to analyze the ANS and cardiovascular using 10 s electrocardiograms.

The results of this study confirm that the greatest physical stress occurs from a momentary fall rather than from an exercise movement. The average SDNN and rMSSD resulting from falls are 0.109 ± 0.114 and 0.124 ± 0.170 s (*p* < 0.05). These results suggest that significant changes in average heartbeat interval are related to the activity of the ANS. However, it was confirmed that there was a significant difference in the activity of the ANS between sitting on a chair and falling to the right and left, which had similar changes in average heartbeat interval, and additional analysis was conducted on this.

**Table 2:** Physical stress action that can occur in daily life

Index	Division	Movement	Method	Rest time(s)
1	Common	Go up the stairs	Continuously	10
2		Go down stairs	Continuously	10
3		Sit on chair	1 time	10
4		Stand up from a chair	1 time	10
5		Lying on the bed	Continuously	10
6		Prostrate oneself on the bed	Continuously	10
7	Exercise	Walk	Continuously	10
8		Sit down and stand up	1 time	30
9		Jump	5 time	30
10	Fall	Falling forward	1 time	30
11		Fall down on your knees	1 time	30
12		Fall backwards	1 time	30
13		Fall to the right	1 time	30
14		Fall to the left	1 time	30
15		Falling out of bed	1 time	30

### Changes in inspiration and exhalation times by movement

**Fig. 4:**
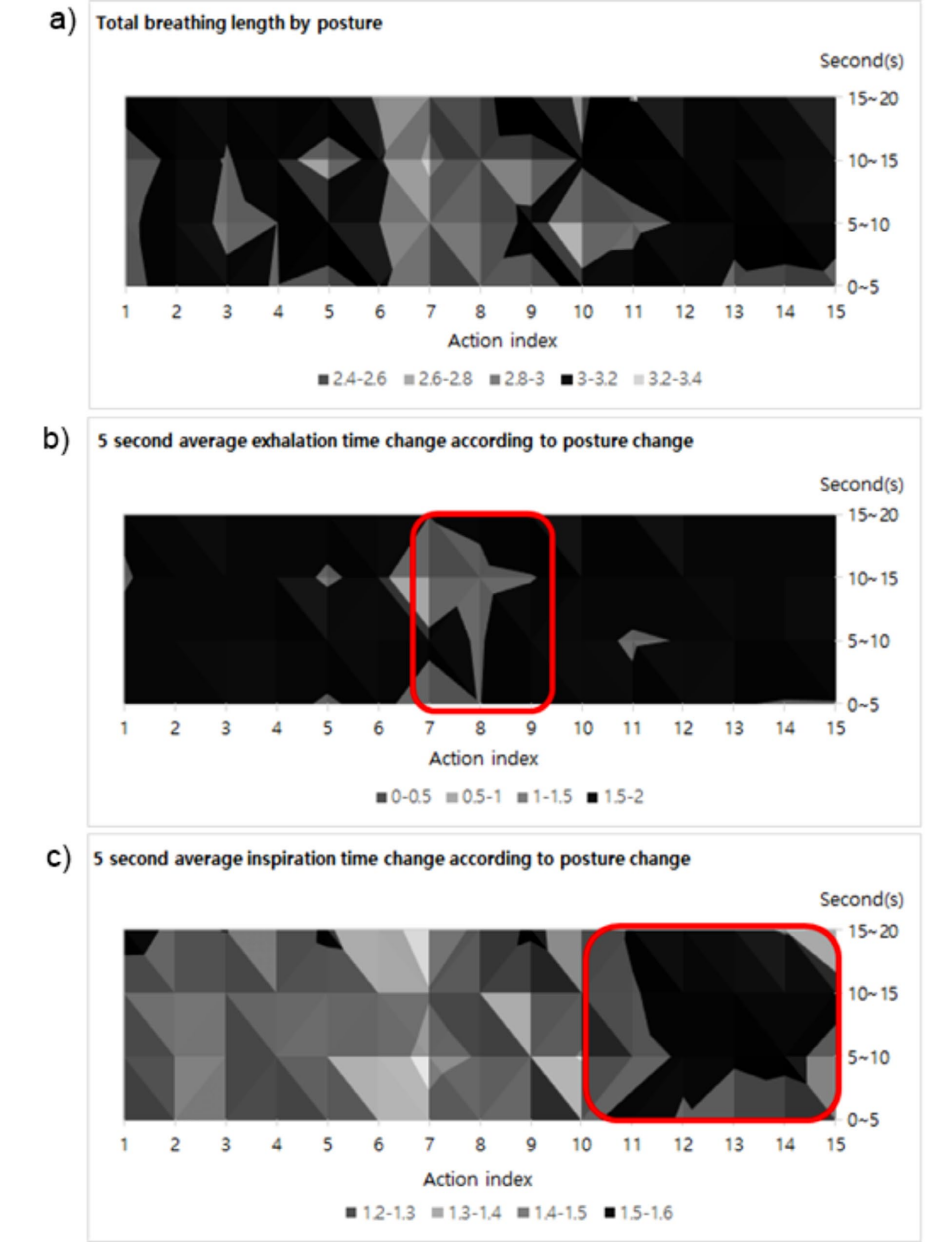
5 s average breathing length for breathing analysis by posture. (**a**) Change in total breathing length for 20 s per action, (**b**) Change in exhalation length for 20 s per action, (**c**) Change in Inspiratory length for 20 s per position

The total length of breathing according to movement is 3.00 ± 0.07 s. Inspiration time was 1.46 ± 0.04 s, and expiration time was 1.53 ± 0.05 s, confirming that the breathing consisted of longer expiration than inspiration. The changes in inspiration and expiration that make up total respiration are shown in Fig. 4. As shown in b of Fig. 4, it can be seen that the expiration time decreases during the exercise movement. Therefore, the overall breathing length shows a significant decrease of less than *p* < 0.05 after 10 s of movement (Fig. 4, a).

However, the important point is the change in inspiration time. There is no change in inspiration time in chair sitting with similar heartbeat interval changes, but it can be seen that the inspiration time increases during the falling movement (Fig. 4, c). Like the exercise movement, the inhalation time significantly increased from 1.46 to 1.51 s after 10 s (*p* < 0.05). Through this, it can be confirmed that there is a correlation between SDNN, rMSSD, and inspiration time shown in result 3 − 1. In other words, it is judged that changes in the cardiovascular system that occur due to a fall induce rapid activation of the ANS and PN systems and increase the inspiratory time of spontaneous breathing to maintain homeostasis.

## Discussion

In this study, we attempted to identify specific breathing methods and changes that stabilize the function of the heart by analyzing changes in spontaneous breathing and the cardiovascular system in an environment of physical stress caused by movements performed in daily life. In general, breathing improves the sensitivity of the baroreflex by stimulating pressure receptors present in the cardiovascular system [30–32]. Changes in the cardiovascular system caused by such stimulation are called RSA. It is related to individual respiratory parameters such as breathing frequency, amplitude, and inspiration and exhalation time, and can change depending on various physical stress situations [33]. In other words, changes in the cardiovascular system induced by the situation are transmitted through the ANS and the respiratory system, and can induce changes in the cardiovascular system through changes in breathing patterns to maintain homeostasis in the human body.

For this reason, early changes in the cardiovascular system and ANS were confirmed through analysis of the cardiovascular system according to the occurrence of movement. The biggest change in the cardiovascular system that occurs with movement is the change in heartbeat interval depending on the position of the heart. When the position of the heart is lowered, such as when sitting down or falling from a standing position, a rapid increase in heartbeat interval occurs. The cause of this is confirmed to be a rapid change in blood pressure and a homeostasis through the baroreflex [34, 35]. A sudden drop in heart position initially causes a rapid rise in blood pressure. When blood pressure rises, heart rate is reflexively reduced to stabilize blood pressure, resulting in an increase in heartbeat interval. Ultimately, homeostasis in the human body is maintained through a decrease in heart rate and an increase in contraction [36]. A medical method that can observe changes in the heart position, blood pressure, and ANS is the head up tilt test [37]. That is, to further observe changes in the ANS, SDNN and rMSSD of heart rate variability were additionally analyzed.

Because action index 3, 13, and 14 in Fig. 3a show similar changes in heartbeat interval, we assumed identical ANS changes. However, the results were different. A rapid increase in ANS activity was observed only in the falling movement. In this study, the cause of this was determined to be head movement. In the case of falling movements, rapid movements and rotations of the head occur. Additionally, the action of falling off the bed at action index 15 also showed a similar increase in ANS. Among them, the vestibular system, which is related to falls, is judged to be the cause [38, 39]. The vestibular system has the greatest correlation with falls and is connected to the cranial nerves, so it can affect the ANS and central nervous system at a faster rate [40].

General ANS activity appears as an increase in physical stress. In the case of rapid breathing, it can increase blood pressure, heart rate and stimulation of the ANS to the heart, but it can also limit the function of expiration flow due to increased work of the respiratory system due to decreased respiratory endurance [41, 42]. These findings may explain the decreased duration of expiration during the exercise movement, as shown in Fig. 3b. However, what is interesting is that the decrease in heart rate and rapid activation of the ANS during a falling movement are opposite situations. As a result, the time of inspiration of breathing increased. Increased inspiratory time causes deep breathing, and deep breathing has various effects on the ANS. deep breathing can affect emotional responses, such as reducing pain intensity and discomfort [43, 44], and can induce changes in the ANS that increase RSA [45–49]. Additionally, it has been confirmed that increased inspiration time during spontaneous breathing can improve mental stress and the cardiovascular system by inducing deep breathing [50, 51]. In other words, it can be seen that among the changes in heartbeat interval caused by blood pressure, in the case of movements that induce rapid changes in the autonomic nervous system, participation in breathing for stability is induced.

Another criterion for determining the relationship between the cardiovascular system and inspiratory time is resistance to breathing. In index 10, 11, and 12 postures of the falling movement, breathing is performed with the chest parallel to the floor, and in index 13 and 14, breathing is performed with the chest perpendicular to the floor. In other words, it was judged that indices 13 and 14 showed greater heartbeat interval changes than indices 10, 11, and 12, which experienced higher resistance to breathing [52–54]. These results may explain the potential relationship between dyspnea and cardiovascular disease due to abnormalities in the autonomic nervous system and cardiovascular system due to aging [55] and decrease in chest wall flexibility [56]. In this study, conditions were not made equal for all participants. It has the limitation of not being able to conduct individualized analyzes such as ANS activity due to diet and smoking, individual breathing rate and muscle mass, and intensity of physical stress. However, this was overcome through quantified average analysis using data obtained from 100 participants measured in various physical stress environments.

## Conclusion

The purpose of this study is to analyze the cardiovascular and respiratory systems that occur due to physical stress, identify effective breathing methods that can affect the cardiovascular system, and propose breathing control methods. In this study, it was confirmed that changes in blood pressure and cardiovascular system that cause significant changes in the ANS can be stabilized by increasing the inspiratory time of breathing. It is expected that these results can be used as a digital treatment that can prevent, manage, and treat potential cardiovascular abnormalities that may occur in a physical stress environment through a breathing method that favors inhalation compared to exhalation time.

### Electronic supplementary material

Below is the link to the electronic supplementary material.

Supplementary Material 1

Supplementary Material 2

Supplementary Material 3
