# Supplementary material for: Analysis of breathing patterns to stabilize cardiovascular changes in physical stress environments : inspiration responds to rapid changes in blood pressure
Source: Biomed Eng Lett. 2024 Apr 10;14(4):813–21. doi: 10.1007/s13534-024-00379-y (PMC11208350; doi:10.1007/s13534-024-00379-y)
Supplement: Supplementary file 1 — Supplementary Material 1 [file 13534_2024_379_MOESM1_ESM.pdf]

연세의료원 세브란스병원 연구심의위원회
Yonsei University Health System, Severance Hospital, Institutional Review Board
서울특별시 서대문구 연세로 50-1 (우) 03722
Tel.02 2228 0430~4, 0450~4 Fax.02 2227 7888~9 Email. irb@yuhs.ac

심 의 일 자	2023년 3 월 20 일
접 수 번 호	2022-3724-002
과제승인번호	1-2023-0006

세브란스병원 연구심의위원회의 심의 결과를 다음과 같이 알려 드립니다.

Protocol No.	
연 구 제 목	홀터 심전계(HiCardi+)를 이용한 낙상 자세별 가속도 센서 변화 측정
연 구 책 임 자	임상희 / 세브란스병원 재활의학과
의 뢰 자	세브란스병원
연구예정기간	2023.03.20 ~ 2024.03.19
지속심의 빈도	12개월마다
과 제 승 인 일	2023.03.20
위 험 수 준	Level Ⅰ 최소위험
심 의 방 법	신속
심 의 유 형	질의답변 + 계획변경
심 의 내 용	- 말씀하신 내용 수정하였습니다.<br>- [변경후] 이영현 - 이해상충(해당없음)<br>- [변경후] 연구 담당자-이영현 삭제<br>- [변경후] 연구 담당자-이영현 추가<br>- [변경후] 대상자 설명문 및 동의서 : : 2. 설명문 및 동의서_ 낙상 임상 연구_230228_1.pdf 삭제<br>- [변경후] 대상자 설명문 및 동의서 : v2 : 2. 설명문 및 동의서_ 낙상 임상 연구_230314.pdf 추가
심 의 위 원 회	제5위원회
참 석 위 원	제5위원회 신속심의자
심 의 결 과	승인
심 의 의 견	-

※ 본 통보서에 기재된 사항은 세브란스병원 연구심의위원회의 기록된 내용과 일치함을 증명합니다.
※ 세브란스병원 연구심의위원회는 국제 임상시험 통일안(ICH-GCP), 임상시험 관리기준(KGCP), 생명윤리 및 안전에 관한 법률을 준수합니다.
※ 연구책임자 및 연구담당자가 IRB위원인 경우, 해당 위원은 위 연구의 심의과정에 참여하지 않았습니다.

연세의료원 세브란스병원

연구심의위원회 위원장

*** 유의사항 ***

1. 세브란스병원 임상연구보호프로그램 규정 준수

세브란스병원에서 수행되는 모든 임상연구는 임상연구보호프로그램 규정을 준수하여야 합니다.
연구책임자께서는 모든 연구관련자들이 규정을 이행할 수 있도록 협조하여 주시기 바랍니다.

2. 이의신청

연구자는 심의결과에 이의가 있을 경우 이의신청을 통해 심의관련 의견제시가 가능합니다.
관련 질의에 대한 의견과 충분한 근거를 제출하여 주시기 바라며, 자료 미흡 또는 근거가 불충분할 경우 연구자에게 추가 자료를 요청할 수 있습니다.

3. 질의답변

승인 통보받지 않은 과제는 연구를 진행할 수 없습니다. 시정승인 또는 보완 결과를 받은 과제는 관련 질의에 대한 답변서와 그에 따른 변경 및 수정된 자료를 심의일로부터 6개월 이내에 제출하여야 합니다.

4. 대상자 동의

IRB 승인을 받은 동의서를 사용하여야 하며, 강제 혹은 부당한 영향이 없는 상태에서 충분한 설명에 근거하여 동의절차가 진행되어야 합니다. 또한, 대상자에게 연구참여여부를 고려할 수 있도록 충분한 시간을 제공하여야 합니다.
대상자 모집공고문을 사용하는 경우에는 모집공고문과 게시방법에 대해 IRB의 사전 승인을 받아야 합니다.

5. 중간보고

관련 법령에 따라 연구의 승인 유효기간은 최대 1년을 넘을 수 없습니다.
IRB가 결정한 심의 빈도에 따라 승인 만료일 최소 6주전에 중간보고를 제출하여 승인 유효기간을 갱신하셔야 합니다.

6. 계획변경

연구진행 시, 대상자 보호를 위해 불가피한 경우를 제외하고 연구절차, 대상자 수 등 IRB로부터 승인받은 내용에 변경이 있을 경우에는 반드시 IRB의 승인을 득한 이후에 적용할 수 있으며, 대상자 보호를 위해 취해진 응급상황에서의 변경도 즉시 IRB에 보고하여 주시기 바랍니다.

7. 안전성 정보 보고

대상자의 안전이나 임상연구에 부정적인 영향을 미칠 수 있는 새로운 정보에 대해 신속히 IRB에 보고하여야 합니다.

8. 종료보고

대상자의 관찰이 종료되고 자료 수집이 완료된 후 20일 이내에 보고하여야 합니다.

9. 결과보고

종료보고 이후, 자료분석 결과에 대해 보고하여야 합니다.

10. 내부점검 시 협조 요청

대상자 보호와 계획서 및 관련 규정 준수를 확인하기 위해 점검을 실시하는 경우, 원활한 점검절차 진행을 위해 연구진행과 관련된 서류를 준비하고 협조하여 주시기 바랍니다.

본
